# The Usefulness of Indocyanine Green in Modern Gynecological Oncology—Analysis, Literature Review, and Future Perspectives

**DOI:** 10.3390/cancers17183081

**Published:** 2025-09-21

**Authors:** Michał Kostrzanowski, Grzegorz Ziółkowski, Agata Mandes, Grzegorz Panek, Michał Ciebiera, Filip Dąbrowski

**Affiliations:** 1 Department of Gynecology and Gynecologic Oncology, Center of Postgraduate Medical Education, Cegłowska 80, 01-809 Warsaw, Poland; grzegorz.ziolkowski@bielanski.med.pl (G.Z.); mandesa@bielanski.med.pl (A.M.); grzegorz.panek@bielanski.med.pl (G.P.); filip.dabrowski@bielanski.med.pl (F.D.); 2 Second Department of Obstetrics and Gynecology, Center of Postgraduate Medical Education, 00-189 Warsaw, Poland; mciebiera@cmkp.edu.pl; 3 Warsaw Institute of Women’s Health, 00-189 Warsaw, Poland

**Keywords:** indocyanine green, fluorescence, compartmental surgery, precision surgery, lymphadenectomy, image-guided surgery

## Abstract

Indocyanine green (ICG) is a fluorescent agent which is characterized by a wide range of applications in the proper visualization of the operating field, differentiation of vital structures, and localization of lesions to be excised. An investigation and overview of novel approaches of indocyanine green in modern gynecological oncology was conducted, including ovarian cancer surgery with its compartmental approach and compartmental surgery in endometrial cancer. Ureteral visualization and perfusion, lymphography, lymph node transfers, or the localization of anastomotic leakage in bowel surgery are examples of applications aimed at reducing the risk of surgical complications and improving the patients’ quality of life. The general use of indocyanine green in lymph node detection, subcategorized and analyzed, is constantly improved and reviewed. A therapeutic approach with macromolecules is being tested in preclinical models. Early results could suggest the future application of indocyanine green not only in broad-sense imaging but also as a cytotoxic agent conjugated with macromolecules. Further studies on the application of indocyanine green in laparoscopy, open surgery, and finally as a curative cytotoxic agent are needed.

## Simple Summary

Indocyanine green (ICG) is a fluorescent agent which is characterized by a wide range of applications in the proper visualization of the operating field. It is particularly important in surgical oncology, where proper visualization translates into the completeness of tumor resection. We reviewed the usefulness of indocyanine green in sentinel lymph node mapping, its application in angiography, and the identification of structures such as ureters or nerves. Preclinical models of using indocyanine green as a cytotoxic agent conjugated with macromolecules were summarized. The use of indocyanine green in this way still requires clinical trials before daily application.

## 1. Introduction

Surgery, in its nature, relies on the proper visualization of the operating field, differentiation of vital structures, and the localization of lesions to be excised. Throughout the history of medicine, surgeons relied only on their own eyes. The discovery of X-rays, the introduction of computed tomography, and ultrasound imaging have significantly facilitated preoperative visualization. The development of chemical contrasting agents and thorough research of fluorescence agents constituted the next milestones in surgical visualization. Fluorescein was one of the first compounds. It was used intravenously in 1948 to enhance the visibility of intracranial neoplasms during neurosurgery [1]. Since that time, a variety of agents have been used for diverse surgical applications [2]. Indocyanine green (ICG), which has a broad spectrum of usefulness in surgery, is one of such compounds.

We decided to review the usefulness of indocyanine green in gynecologic cancer treatment and its possible applications in gynecologic oncology in the future. We searched electronic databases such as Google Scholar and PubMed. We focused only on the application of pure indocyanine green alone, or where its use was compared to other substances in the treatment of cancer of the female reproductive tract. The keywords used in the title search were “indocyanine” or “near infrared” or “fluorescence” with “gynecologic” or “gynecological”. We retrieved 48 results from PubMed. Out of those, we searched, analyzed, and included applications of indocyanine green in the treatment of gynecologic tumors.

## 2. Physical Characteristics

Indocyanine green, developed in 1955 by Kodak Research Laboratories [3], has been used in retinal angiography since the early 1970s [4]. ICG is a tricarbocyanine dye that belongs to the group of cyanine dyes. It is soluble in water [5]. In vivo, ICG binds to lipoproteins, whereby the structure of the protein is not altered, which is a sign of nontoxicity [6]. This dye does not have any metabolites; it is released by the liver into the bile juice [7]. The concentration used for in vivo retinal and choroidal angiography is 20–25 mg/mL, injected into a peripheral arm vein [8]. A concentration of 0.5 mg/kg of body weight is recommended for studies of hepatic function. No toxic effects were observed in humans at a dose of 5 mg/kg of body weight [9]. The bound indocyanine green may be excited with 805 nm light and it emits a wavelength range from 810 nm to 875 nm [10]. Then, using near-infrared ray imaging, we may see the structures colored by ICG [11]. Such wavelengths are invisible to the naked eye, and they pass quite well through the surrounding tissue. It is caused by the relatively low adsorption of light by hemoglobin or water. Therefore, images of structures below 5 mm from the tissue surface may be seen [10].

The basic concept of cancer surgery includes the complete resection of cancer tissue within the primary tumor and metastases, including metastatic lymph nodes. In cancer surgery, the mapping of sentinel lymph nodes is the main domain of the usefulness of labeling substances, where the potential spread of tumor tissue may be detected [12]. The ability of indocyanine green to form macromolecules with lipids in the blood circulation has allowed the use of indocyanine green for applications other than mapping lymph nodes. The macromolecules tend to accumulate in cancer tissue due to increased vascular permeability and reduced drainage. This could be observed in solid tumors and is called the enhanced permeability and retention (EPR) effect [13]. As regards the processes in the modified tumor tissue, we know that peritoneal metastases are characterized by the formation of abnormal, large new vessels with convolutedness, which may be detected over the metastatic deposits. This phenomenon is also seen in age-related macular degeneration, where ICG administered intravenously allows for the detection of choroidal neovascularization [14]. Both of these characteristics are widely used in gynecologic oncology, depending on the type of cancer of the female genital tract.

In gynecologic cancers, indocyanine green is routinely used in early-stage cervical and endometrial cancer. In these two diseases, its use comes down to sentinel lymph node (SLN) mapping and has been accepted by the National Comprehensive Cancer Network and the European Society of Gynecologic Oncology guidelines [15,16].

## 3. Lymphadenectomy and Compartmental Surgery in Ovarian Cancer

In ovarian cancer, detecting lymphatic drainage and lymph nodes is crucial in the surgery of early-stage disease, FIGO I, and FIGO II, when bilateral pelvic and para-aortic lymph node dissection is generally recommended [17]. Two major pathways and one minor pathway of the lymphatic drainage of the ovary were confirmed immunohistochemically in one study. The first pathway drains via the infundibulopelvic ligament to the para-aortic region on the left side and the paracaval region on the right side. The second major pathway drains via the proper ligament of the ovary to the obturator fossa, and the third pathway, a minor one, drains via the round ligament to the inguinal region [18]. The most common locations of lymph node metastases in ovarian cancer are the para-aortic/paracaval area (50%), the pelvic area (20%), and both the para-aortic/paracaval and pelvic areas (30%) [18]. Positive lymph nodes with the disease limited to the ovary are found in 10–15% of the patients, while lymphatic metastases occur in 64–67% of the patients in whom the disease spread to the abdomen [19].

The use of indocyanine green seems to be promising at the early stages of the disease when lymphadenectomy is recommended. In the literature, we identified three studies describing 43 patients with ovarian cancer who underwent sentinel lymph node mapping using indocyanine green alone [20,21,22]. In those studies, the injection sites were the hilum of the ovary, the proper ligament of the ovary, or the suspensory ligament of the ovary. The concentration of ICG solution was 1.25 mg/mL and the volume administered varied between 0.5 mL and 2.0 mL. The time between injections and detection varied and ranged between real-time detection and 20 min. A minimally invasive surgery was performed in all cases. The detection rate was 90.5%. When using the combination of indocyanine green and Tc99m nanocolloid, the detection rate reached 100% [23]. The detection rate calculated from those studies showed the feasibility of indocyanine green in mapping sentinel lymph nodes in ovarian cancer. However, we should approach the results with caution due to the limited number of studies and different injection sites.

A new interesting approach was identified in the literature, i.e., targeted compartmental lymphadenectomy which was used in early-stage ovarian cancer. As mentioned above, there are two main pathways of lymphatic drainage from the ovary. The ovarian drainage occurs via the infundibulopelvic ligament known as the ovarian mesonephric pathway [24] and the second pathway occurs via the proper ligament of the ovary, known as the Müllerian uterine pathway [18]. Indocyanine green injection into the uterine corpus made it possible to visualize the Müllerian uterine pathway and ovarian mesonephric pathway equally effectively [25]. In two cases, using this technique of labeling ovarian lymphatic drainage, the authors removed the ovary with the cancer, its lymphatic vessels, and at least the first two sentinel nodes in each channel en bloc, i.e., to the pelvic part of lymphatic drainage [24] and the para-aortic drainage, respectively [26]. The procedures were consistent with the ontogenetic approach with respect to the locoregional control [27]. Therefore, compartmental lymphadenectomy rather than systematic lymphadenectomy could be the new standard in early ovarian cancer surgery [24].

## 4. Vulvar Cancer

**Figure 1:**
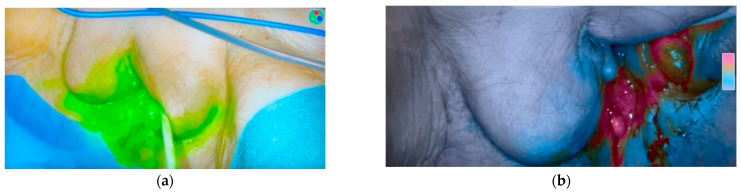
Vulva after the administration of indocyanine green in two fluorescence modes: (**a**) color mode; (**b**) color segmented fluorescence (CSF) mode.

The applications of indocyanine green in lymph node mapping were also found in vulvar carcinoma. The lymphatic drainage depends on the location of the primary tumor. If located in the anterior one-third of the vulva, the tumor drains to the superficial inguinal nodes and the mons; tumors located in the posterior two-thirds of the vulva drain to the superficial and deep inguinal nodes. This was confirmed after long-term observations while performing en bloc resections of vulvar cancer [28,29,30]. Nowadays, sentinel lymph node mapping is routinely used in the early stages of vulvar cancer. This is recommended in cases of unifocal tumors smaller than 4 cm, without suspicious inguinofemoral nodes [31]. In the mapping of sentinel lymph nodes of vulvar cancer, the combination of blue dye and Tc99m nanocolloid is used in the first place. It was the main method in the large GROningen International Study on Sentinel nodes in Vulvar Cancer V (GROINSS-V), whose authors confirmed the validity of sentinel lymph node dissection in patients with early-stage vulvar cancer [32]. There is a growing interest in the use of indocyanine green/Tc99m nanocolloid instead of the combination of blue dye and Tc99m nanocolloid. A randomized controlled trial was conducted in a group of 24 patients. It revealed that the intraoperative visual detection of sentinel lymph nodes using ICG-99mTc-nanocolloid was superior compared to 99mTc-nanocolloid and blue dye in patients with early-stage vulvar squamous cell carcinoma. However, the rate of successful procedures of sentinel lymph node dissection was comparable in both groups [33]. A meta-analysis of studies published between 2010 and 2020 showed that the detection rate of sentinel lymph nodes was higher when using indocyanine green alone or in combination with lymphoscintigraphy over blue dye [34]. A series from Memorial Sloan Kettering Cancer Center revealed that 100% of groins were mapped using the ICG-99mTc-nanocolloid compared to 91.8% of groins that were mapped with 99mTc-nanocolloid and blue dye. When ICG was used alone, the detection rate was 96.3% as mapping failed in one groin [35]. Prader et al. demonstrated that the detection rate of sentinel lymph nodes increased with the duration of the study, which means that experience and training may have a significant impact on the final outcome [36]. The imaging protocol for the use of indocyanine green in early-stage vulvar cancer is still to be accepted as a standard of care (Figure 1) [31].

**Figure 2:**
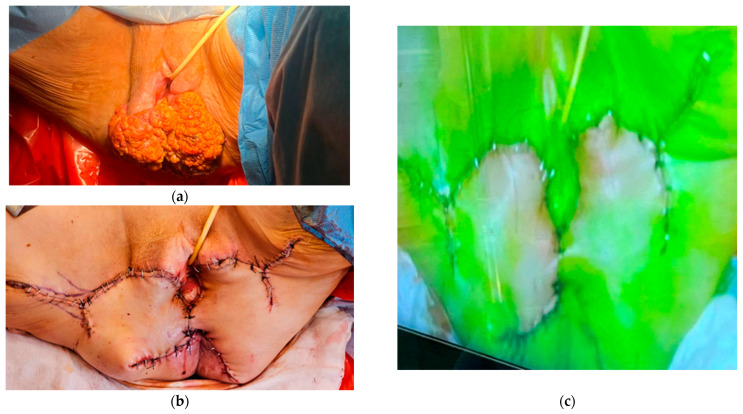
The use of indocyanine green in the evaluation of skin flap perfusion after vulvar cancer resection: (**a**) vulvar cancer location; (**b**) perineum after vulvar flap reconstructive surgery in the same patient; (**c**) the assessment of skin flap perfusion in the fluorescence mode with the use of indocyanine green.

In patients with vulvar cancer, the usefulness of indocyanine green is not limited to sentinel lymph node mapping. In such patients, radical vulvectomy and a subsequent vulvar flap reconstructive surgery are often performed. The intraoperative flap vitality assessment is essential for avoiding surgical complications later on [37]. Vulvar flap evaluation using angiography with indocyanine green as a tracer would be a feasible method. A study conducted in a group of 15 patients showed dye uptake on the surgical edge of the flap, thereby confirming vulvar flap viability. No complications, such as a surgical infection, dehiscence, or necrosis were observed later [38]. Additionally, flap skin viability assessment in real-time surgery allowed the identification and removal of a low vascular perfusion area to avoid postoperative complications (Figure 2) [39].

## 5. Cervical Cancer

Pelvic lymph nodes, beyond the uterus and upper vagina, are some of the sites where cervical cancer spreads [40]. The main prognostic factors are the pathological tumor type including HPV status, margin status, depth of cervical stromal invasion, presence of distant metastases, lymphovascular stromal invasion, and the FIGO stage including nodal involvement [41]. The reported survival rates for patients with stage IB cervical cancer are between 83% and 90% [42]. However, when positive lymph nodes are encountered, the survival rate is 50% [43]. A literature search revealed several papers describing indocyanine green in sentinel lymph node mapping in cervical cancer. In those studies, patients with cervical cancer accounted for 5% to 10% of the participants, and ICG use turned out to be a safe, feasible method for sentinel lymph node detection [44,45]. Ruscito et al. suggested that the efficacy of ICG in SLN detection was comparable with that of methylene blue and Tc99m nanocolloid [46], whereas Diab, Jewell et al. and Ulain et al. concluded that bilateral detection rates with ICG were superior to those obtained with other tracers [45,47,48]. A randomized, phase 3, multicenter, non-inferiority trial (FILM) revealed that more sentinel nodes were detected with ICG than with isosulfan blue dye in cervical and uterine cancer, with no difference in the pathological confirmation between these two substances. The patients with cervical cancer accounted for 4% of all patients in whom ICG was used [49]. Other authors compared a group of 76 patients in whom a radiocolloid tracer with blue dye was used and a group of 68 patients administered indocyanine green. The detection rates of sentinel lymph node mapping were 96% and 100% for Tc99m nanocolloid with blue dye and indocyanine green, respectively. Bilateral mapping was achieved in 98.5% for indocyanine green and 76.3% for Tc99m nanocolloid with blue dye [50]. The numbers quoted above appear to favor the use of indocyanine green in the surgical treatment of early-stage cervical cancer. However, the studies used laparoscopic or robot-assisted approaches. In 2024, the final analysis of a prospective non-inferiority randomized trial (Laparoscopic Approach to Cervical Cancer [LACC]) was published. As regards a group of patients with stages IA1–IB1 of cervical cancer, the overall survival at 4.5 years was 90.6% in the minimally invasive approach group compared to 96.2% in the laparotomy approach group. The risk of death due to cervical cancer was over 2.5-times higher in the minimally invasive group [51]. These are strong data confirming that an open approach should be the standard of care. However, techniques using near-infrared technology are approved in laparoscopy.

## 6. Endometrial Cancer

Sentinel lymph node mapping in endometrial cancer remains the main application of indocyanine green in modern gynecologic oncology. Sentinel lymph detection appeared a viable alternative to comprehensive lymphadenectomy in endometrial cancer staging [52]. In the FIRES multicenter, prospective, cohort study, the sensitivity of sentinel lymph node mapping was 97.2% in nodal metastasis identification. The negative predictive value was 99.6%, and sentinel lymph node staging could successfully replace lymphadenectomy in the procedure of endometrial cancer staging [53]. The intracervical site of injection makes it possible to obtain high bilateral sentinel lymph node visualization [54]. A needle is inserted at the 3 o’clock and 9 o’clock position of the cervix, then indocyanine green solution is injected into the cervical stroma, and the course of indocyanine dye is visualized using near-infrared technology [55]. The detection rate of sentinel lymph nodes using ICG could reach the level of 90.9% [56] and was higher than the detection rate when using blue dye with Tc99m nanocolloid which ranged from 27% to 69% [57]. One study showed that the detection rate when using only blue dye ranged from 77% to 94% [58]. Nevertheless, the intracervical administration of indocyanine green constitutes the preferred detection technique [52]. In the FILM study, mentioned in the section of cervical cancer, the identification rate of one or more sentinel lymph nodes was 96% when using indocyanine green only. When using blue dye only, it was 74%. The identification of bilateral sentinel lymph nodes was at the level of 78% and 38%, respectively [49]. The detection of sentinel lymph nodes during surgery decreased the total number of resected pelvic lymph nodes and was associated with a lower risk of intraoperative complications. Additionally, in postoperative observations, no complications were seen during 30 postoperative days and the risk of lower-limb lymphedema was not increased [59]. In the Surveillance, Epidemiology, and End Results (SEER) database analysis from 2023 conducted in patients with grade 1-2 endometrioid-type endometrial cancer, longer survival rates were observed in the group of sentinel lymph nodes. In patients with high-grade endometrial cancer (non-endometrioid-type, grade 3 endometrioid-type), no differences in survival were observed between the group with sentinel lymph nodes and the group with lymph node dissections [60]. It is worth mentioning that pelvic lymph nodes will not be visualized or all morphologically suspicious lymph nodes will not be stained in all cases. However, regardless of mapping, all suspicious lymph nodes have to be resected according to the sentinel lymph node removal algorithm [61].

In endometrial cancer, the use of indocyanine green has also been applied in embryologically defined compartmental surgery. After the injection of indocyanine green into the uterine corpus, Kimmig et al. reported the visualization of two main pathways of lymphatic drainage: the first pathway—to the lymph nodes along the external and iliac vessels; the second pathway—to the para-aortic nodes. Subsequently, intermediate/high-risk patients underwent radical hysterectomy with peritoneal mesometrial resection and with pelvic and para-aortic lymphadenectomy. Low-risk patients underwent simple extrafascial hysterectomy. Locoregional recurrence was observed at the level of 2.9%. The authors concluded that the visualization of the embryological compartments with lymphatic drainage during real-time surgery seemed to be a promising method in endometrial cancer surgery, especially in the prevention of the locoregional recurrence of the disease [62].

## 7. Ureteral Visualization

Indocyanine green has an additional application, not mentioned above, in gynecologic cancer surgery. Ureters, lying in the retroperitoneal space, are vulnerable to intraoperative injury. The most vulnerable site of iatrogenic injury is the place where the ureter passes through the medial aspect of the paracervix with the risk of injuries varying between 0.5% and 5%. The operating surgeon’s unawareness of the proximity of the ureters could lead to intraoperative injuries, especially when the procedure is more complex and the anatomy is distorted [63]. Many such injuries may not be recognized intraoperatively [64]. The use of near-infrared imaging technology could help to identify the ureter during surgery. The first use of indocyanine green in the visualization of ureteral strictures during robot-assisted surgery was reported in 2013 [65]. This technique could be applied successfully both in laparoscopy and laparotomy. First, standard cystoscopy was performed. During cystoscopy, both ureteric orifices were localized, then ureteric catheters were advanced into the ureters and the ICG solution was injected. Subsequently, the catheters were removed and ureters could be localized immediately using near-infrared imaging [66]. The authors of a pilot study identified 96.5% ureters in a group of 120 cases. They observed no complications related to cystoscopy and no significant increase in surgery duration [67]. The identification of the ureters by retrograde cystoscopy with ICG infusion is a safe and effective method which does not extend the duration of the surgical procedure. Some authors analyzed the outcome of bladder-preserving ureteral reconstruction in cancer surgery. The major complications that occurred were associated with a history of pelvic irradiation. They concluded that personal surgery should be planned in such cases, especially with a history of adjuvant radiation therapy [68]. This is the reason for the use of indocyanine green in patients with a higher risk of complications [69].

## 8. Nerve Visualization

Radical pelvic surgery is often associated with iatrogenic neural damage that leads to severe motor, sensory, and autonomic dysfunctions such as urinary incontinence, diarrhea, constipation, and sexual dysfunction [70]. Statistically, the incidence of genitofemoral and obturator nerve injuries occurs in 3.5% and 1% of cases, respectively [71,72]. The protection of vital structures, including pelvic nerves, helps to reduce the risk of complications and is essential in achieving the best patient prognosis [73,74]. Intraoperative nerve visualization seems to be crucial for reducing iatrogenic neural injuries. However, it is still difficult with the naked eye even for the most experienced surgeons [75,76]. Novel nerve visualization methods are under investigation [77]. The authors of a prospective study used indocyanine green to identify nerve courses in the pelvis. They administered indocyanine green intravenously 24 hours preoperatively and tried to visualize nerve courses in real-time surgery. The bilateral identification rates for the obturator, genitofemoral, and hypogastric nerve were 100%, 93.7%, and 81.0%, respectively. The results showed that fluorescent agents might be safely and effectively used in pelvic nerve identification [78]. More research is needed to confirm the results of surgeries with fluorescence nerve imaging and the risk of complications in this type of procedure.

## 9. Lymphography and Lymph Node Transfer

Indocyanine green is also applied in lymphography and lymph node transfer in the treatment of complications after cancer surgery. Lymphedema may be defined as an interstitial fluid collection which leads to tissue swelling, inflammation, fibrosis, and fat hypertrophy [79]. As regards patients with gynecological cancers after pelvic node dissections, lower-limb lymphedema was found to occur in 2.4% to 41% of cases [80]. Clinically, lymphedema ranges from adipose deposition-dominant to fluid-dominant [69]. Typically, patients with the fat hypertrophy-dominant type are candidates for liposuction [81], whereas patients with the fluid edema type are treated with lymphovenous anastomosis [80] or with vascularized lymph node transfer [82]. The first step to assess the lymphatic drainage and degree of lymphedema progress is the use of indocyanine green in lymphography. The subcutaneous injection of indocyanine green at the foot makes it possible to obtain subcutaneous lymphatic drainage from the foot to the groin in fluorescence [83]. After the visualization of lymphatic drainage, performing anastomosis between lymphatic vessels and venules allows the redirection of the lymph flow beyond the stenosis site and draining the excess of the fluid from the lymphedematous limb [83]. When early-stage lymphedema is identified in relatively healthy and non-obese patients, vascularized lymph node transfer should be considered [82,84]. In this procedure, en bloc lymph nodes are transferred into the affected site with the surrounding healthy tissue. Then, arterial and venous anastomosis is performed [84]. The typical donor sites include lymph nodes from the inguinal region, axilla, omentum, submental region, and lateral thoracic and supraclavicular nodes [82]. The transplanted nodes actively secrete lymphangiogenic factors, which facilitate the regeneration of new lymphatic vessels [85]. Vascularized lymph node transfer is a good method for improving lymphatic function. Lymphovenous bypass is also effective, especially in patients with lower stages of lower-extremity lymphedema. Lymphovenous bypass is less invasive and requires shorter hospitalization of the patients [86].

## 10. Bowel Surgery

At diagnosis, ovarian cancer is at the advanced stage of the disease (FIGO III or FIGO IV) in 65% to 75% of patients. When qualified for surgery, the patient undergoes debulking cytoreduction. The completeness of cytoreduction is still the main prognostic factor for such patients [87]. If the disease involves the rectosigmoid colon at advanced stages, the resection of the sigmoid colon is mandatory to achieve optimal cytoreduction [88]. In this type of procedure, bowel resection is performed in the majority of patients. A multicenter analysis showed that 64% to 79% of patients underwent bowel resection [88,89]. Anastomotic leakage is one of the major complications of bowel surgery with a negative impact on the overall prognosis [90]. The main risk factors that were found to increase the risk of anastomotic leakage included advanced age, multiple bowel resections, low body mass index, and low serum albumin level [90,91,92]. Near-infrared angiography with the use of indocyanine green in the intraoperative visualization of anastomosis might be a promising tool in identifying the risk of anastomoses and reducing the occurrence of anastomotic leakage [93]. The authors of a systematic review confirmed that indocyanine green fluorescence angiography significantly reduced the risk of anastomotic leakage. Additionally, the technique did not extend the operative time and did not increase the risk of postoperative complications [94]. In a multi-institutional (PILLAR II) study, the successful imaging of anastomosis was obtained at the level of 98.6% and the anastomotic leakage rate was 1.4% [95]. In another study (PILLAR III), the anastomotic leakage rate was 9.0% in the fluorescence group of patients and 9.0% in the standard group (*p* = 0.34). Although the trial was terminated before reaching the target number of 450 patients due to a low accrual rate, no difference in anastomotic leakage occurred between perfusion and the control arm in a group of 347 recruited patients [96]. Alekseev et al. demonstrated a lower risk of anastomotic leakage for low anterior anastomosis in the group of fluorescence angiography and no difference between the perfusion group and control group in high anastomoses (9–15 cm from the anal verge) [97]. A retrospective study concerning the surgery of gynecologic malignancies revealed one anastomotic leakage in the total number of 100 anastomoses assessed with fluorescence angiography [98]. Promising results were obtained when proctoscopy was used in the assessment of anastomotic perfusion with near-infrared angiography. A 60% reduction in the odds of anastomotic leakage and postoperative abscess occurrence was noted in a cohort of patients with the NIR visualization of the anastomosis [99]. The majority of studies mentioned above mainly concentrated on patients with colorectal cancer or inflammatory bowel disease. Patients operated on due to female reproductive tract cancers receive different adjuvant treatment and the surgical technique also varies. Prospective cohort studies are still needed in patients undergoing gynecologic surgeries for the objective evaluation of the validity of indocyanine green use in the perfusion assessment of anastomosis.

## 11. Therapeutic Applications

**Figure 3:**
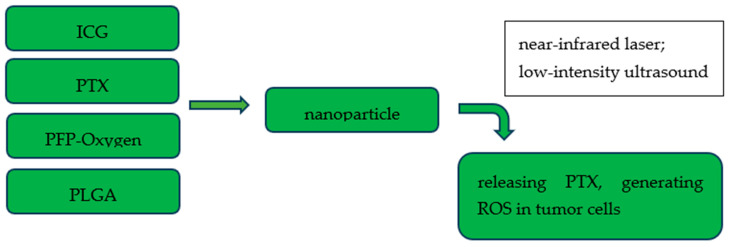
Scheme of paclitaxel, indocyanine green- and oxygen-loaded nanoparticles and their therapeutic applications. PLGA: poly(DL-lactide-co-glycoclic acid); PFP-oxygen: perfluoro-n-pentane-carried oxygen; PTX: paclitaxel; ICG: indocyanine green; ROS: reactive oxygen species.

In near-infrared technology, indocyanine green has a wide range of applications in the imaging of lymph nodes and cancer lesions or as a method of perfusion assessment. There is an increasing number of reports of preclinical studies concerning therapeutic applications of indocyanine green in gynecologic cancers, especially in ovarian and cervical cancer. The combination of nanoparticles and fluorescence agents has the evolving potential in diagnosis and treatment [100,101]. Chen et al. created folate receptor-targeted nanoparticles loaded with indocyanine green and carrying oxygen. After targeting ovarian cancer cells overexpressing folate receptors, the therapeutic effect was achieved by generating reactive oxygen species in combination with indocyanine green as a sensitizer [102]. Photodynamic and sonodynamic therapy using oxygen- and indocyanine green-loaded nanoparticles exhibited a potential cytotoxic effect in preclinical models [103]. Studies on nanomolecules with ICG and chemotherapeutic agents, such as doxorubicin, oxaliplatin, and taxol, also yielded satisfactory results in terms of imaging and cytotoxic effects (Figure 3) [100,104,105].

A combination of the therapeutic effects of nanoparticles and immunotherapy has also found its place in gynecologic oncology. Some authors fused a murine-derived ID8 ovarian cancer cell membrane with a red blood cell membrane creating a hybrid biomimetic coating (IRM). Then, hybrid IRM camouflaged indocyanine green (ICG)-loaded magnetic nanoparticles (Fe3O4-ICG@IRM) were synthesized for the combination therapy of ovarian cancer. Nanoparticles prepared in this way could effectively target tumor cells and showed synergistic photothermal therapy, resulting in the release of whole-cell tumor antigens via photothermal-induced tumor necrosis, which enhanced antitumor immunotherapy for the primary tumor and metastatic tumor by activating CD8+ cytotoxic T cells and reducing regulatory Foxp3+ T cells [106].

The use of indocyanine green in the photothermal therapy of cervical cancer is another example of its use as a chemical part of nanoparticles. In this kind of treatment, photothermal agents under light irradiation kill tumor cells through thermal apoptosis. In one study, indocyanine green was encapsulated by polydopamine (PDA) to form ICG@PDA nanoparticles. This substrate was subsequently modified with a methoxy polyethylene glycol amine (mPEG2000-NH2) to form ICG@PDA@PEG nanoparticles. Afterwards, those nanoparticles were injected into the tumor tissue of the cervical tumor-bearing mice. Under near-infrared 808 nm laser excitation, the tumor of mice was significantly reduced due to the photothermal effect. The authors confirmed that those ICG nanoparticles had a promising potential in antitumor photothermal therapy [107].

Indocyanine green conjugated with recombinant human chorionic gonadotropin (hCG) protein is another substance that uses indocyanine green as a conjugate, although not as a direct therapeutic agent. This substance was found to target early follicles; then, follicular development and ovulation in the near-infrared II window (NIR-II, 1000–1700 nm) could be monitored. From the oncological point of view, NIR-II imaging clearly targeted ovarian tumors and showed micro-metastatic lesions. Those properties could be effectively used for monitoring tumors in vivo or in guiding surgical resections [108].

Some limitations related to the use of ICG as a chemical substance may be listed, i.e., limited photostability, a moderate fluorescence quantum yield, a high plasma protein binding rate, and a tendency to aggregate in an aqueous solution [109]. These shortcomings may be overcome by incorporating ICG into the structure of nanoparticles. Research showed that the synthesis of nanoparticles with ICG and hyaluronic acid allowed the enhancement of the stability of ICG and exhibited cytotoxic effects and intracellular uptake through CD44 receptor-mediated endocytosis in cervical cancer cells [110].

The studies mentioned above focused on the usefulness of indocyanine green in creating nanoparticles whose cytotoxic effect was confirmed via generating reactive oxygen species, releasing chemotherapeutics, immunotherapy, or photothermal therapy. Although all the studies were performed in preclinical models, the satisfactory results should prompt the use of nanoparticles with indocyanine green in clinical trials and apply their properties in everyday treatment.

## 12. Discussion and Future Perspectives

The discovery of fluorescent properties of chemical compounds allows their use as labeling substances. In gynecologic oncology, labeling is effectively used in mapping lymph nodes, angiography, or in the identification of vital structures during real-time surgery. The fundamental application of indocyanine green in gynecologic cancer surgery is still related to its use in sentinel lymph node mapping in cervical and endometrial cancer. This translates into the higher accuracy of sentinel node detection, less need for full pelvic lymphadenectomy, and a reduction in the total number of resected pelvic lymph nodes. It is also associated with a lower risk of intraoperative complications. Nowadays, when a significant number of procedures performed in these two cancers are robot-assisted, indocyanine green still remains the main tracer in sentinel lymph node detection [111,112]. Vulvar tumor is another diagnosis in which sentinel lymph node mapping is used successfully in lymph node detection. Using near-infrared cameras allows the successful identification of sentinel lymph nodes during real-time surgery in the inguinal region. Today and in the near future, trends will be based on replacing blue dye and Tc99m nanocolloid in favor of indocyanine green with a radiocolloid or alone. The key scheme will involve the preoperative assessment of the nodal status using ultrasonography (US), computed tomography (CT), magnetic resonance imaging (MRI), or fluoro-D-glucose positron emission tomography/CT (FDG-PET/CT) with the intraoperative assessment by using near-infrared cameras [113].

Surgery in the inguinal region and pelvic lymph node dissection are correlated with postoperative complications such as lymphedema. Using near-infrared technology, we could trace lymphatic drainage from the lower limbs. Afterwards, vascularized lymph node transfer or lymphovenous bypass could be performed in the treatment of lymphedema. The treatment of lymphedema by surgery together with drugs affecting the immune response is an innovation. Some authors showed that using tacrolimus decreased dermal and subcutaneous T-cell infiltration and tissue fibrosis after lymphatic injury. Such changes prevented the development of lymphedema and could reverse pathologic changes when lymphedema occurred. The study was performed on an animal model but the results may translate into the registration of the drug for this indication [114].

A significant part of the use of indocyanine green is to prevent and reduce the number of complications in gyne-oncologic surgery. This is related to its usefulness in ureteral and nerve visualization and its increasingly widespread use in the assessment of anastomotic leakage in bowel surgery. Recently, some authors have once again presented strong evidence to confirm that indocyanine green fluorescence angiography (ICGFA) colorectal perfusion assessment was associated with lower anastomotic leakage (AL) rates. Based on 45 studies comprising 14333 patients, the AL rate was 6.8%, 4.5% with ICGFA, and 8.5% without it (OR:0.47, *p* < 0.001) [115]. These data should be approached with caution as the study was not performed only on gynecologic oncology procedures. Nevertheless, the main idea of cytoreductive surgery and colorectal resections remains the same, independently of the primary indications. As regards future perspectives, artificial intelligence (AI) will be the next step in improving visualization and perfusion assessment in bowel surgery. In one study, a deep learning model was confirmed as an effective method in the prediction of poor perfusion after surgical anastomosis. This algorithm demonstrated generalizability among ICGFA users from different institutions. Therefore, further blinded studies are needed to establish artificial intelligence in everyday, common use [116].

Studies introducing indocyanine green as a part of nanoparticles have provided promising results in new branches of cancer treatment methods, such as immunotherapy or photothermal therapy. The limitations of ICG resulting from physicochemical properties were also overcome. Data from this research are still limited to single animal experiments. However, the primary results and the constant need to modernize cancer treatment methods should lead to the development of clinical trials with indocyanine green combined with nanoparticles.

## 13. Conclusions

Fluorescent characteristics of indocyanine green have allowed its widespread use in modern gynecologic cancer surgery. Real-time visualization of the essential structures in near-infrared rays imaging constitute a clear benefit for surgeons. This is particularly important in surgical oncology where proper visualization translates into the completeness of tumor resection. The applicability of indocyanine green in various types of gynecologic cancers was demonstrated in Table 1.

We outlined a review of the usefulness of indocyanine green in sentinel lymph node mapping and showed the broad spectrum of its application in angiography and the identification of other vital structures such as ureters or nerves. The novel cytotoxic approaches of indocyanine green still require clinical trials before everyday application.

## Figures and Tables

**Table 1:** Summary of the usefulness of indocyanine green in gynecologic cancers.

Type of Cancer/Indications.	Applications of Indocyanine Green.
ovarian cancer	sentinel lymph node mapping; targeted compartmental lymphadenectomy (labeling ovarian lymphatic drainage)
vulvar cancer	sentinel lymph node mapping at the early stages of vulvar cancer
cervical cancer	sentinel lymph node mapping
endometrial cancer	sentinel lymph node staging; embryologically based compartmental surgery
lymphedema	lymphography; lymph node transfer
ureteral visualization	localization of ureters using near-infrared imaging; easier identification of ureters in patients with a high risk of complications
nerve visualization	pelvic nerve identification to lower the risk of nerve injuries
bowel surgery	indocyanine green fluorescence angiography colorectal perfusion assessment; reduction in the odds of anastomotic leakage
nanoparticles with indocyanine green (preclinical models)	photodynamic, sonodynamic therapy; immunotherapy, photothermal therapy, creating ICG particles to enhance the stability of ICG

## Data Availability

The original contributions presented in this study are included in the article. Further inquiries can be directed to the corresponding author(s).
